# Clinical value of myocardial performance index in patients with isolated diastolic dysfunction

**DOI:** 10.1186/s12947-019-0167-x

**Published:** 2019-08-13

**Authors:** José Maria Gonçalves Fernandes, Benício de Oliveira Romão, Ivan Romero Rivera, Maria Alayde Mendonça, Francisco de Assis Costa, Margareth de Souza Lira Handro, Orlando Campos, Ângelo Amato V. De Paola, Valdir Ambrósio Moisés

**Affiliations:** 1 Faculty of Medicine, Federal University of Alagoas, Av Lourival Melo Mota, sn, Tabuleiro dos Martins, Maceió, 57072-900 Brazil; 2 Federal University of Alagoas, Arapiraca, Brazil; 3 Harmony Medical Center, Maceió, Brazil; 4 Federal University of São Paulo, São Paulo, Brazil

**Keywords:** Echocardiography, Doppler, Ventricular function, left, Heart failure, diastolic, Hypertension

## Abstract

**Aims:**

The Doppler-derived myocardial performance index (MPI) has been considered as a diagnostic and prognostic Doppler marker for many different clinical conditions. The purpose of this study was to determine the diagnostic accuracy of traditional Pulsed-wave Doppler (PWD-MPI) and Pulsed-wave tissue Doppler imaging (TDI-MPI) and the degree of agreement between these methods in patients with grade-I diastolic dysfunction (DDI) and a normal ejection fraction.

**Methods:**

Forty-seven consecutive ambulatory patients with DDI were compared to 51 healthy subjects with normal echocardiograms. All subjects underwent measurement of time intervals and MPI with PWD and pulsed TDI.

**Results:**

TDI-MPI and PWD-MPI were significantly higher in patients with DDI than in control subjects: 0.49 ± 0.14 vs. 0.40 ± 0.09 (*P* < 0.001) and 0.45 ± 0.11 vs. 0.37 ± 0.08 (*P* < 0.001), respectively. Cutoff values of TDI-MPI > 0.42 and PWD-MPI > 0.40 identified DDI subjects, with sensitivities of 74 and 64%; specificities of 61 and 69%; positive likelihood ratios of 1.9 and 2.0; and negative likelihood ratios of 0.42 and 0.53, respectively; no significant difference was noted between the areas under the ROC curves of TDI-MPI and PWD-MPI (*P* = 0.77). Bland-Altman plots showed wide limits of agreement between these indices: − 0.17 to 0.23 in healthy subjects and − 0.24 to 0.32 in DDI patients.

**Conclusion:**

PWD-MPI and TDI-MPI showed poor clinical agreement and were not reliable parameters for the assessment of left ventricular diastolic function.

## Introduction

Initial diastolic dysfunction detected by Doppler echocardiography is an independent risk factor for the development of heart failure and all-cause mortality, even in asymptomatic patients [1]. The myocardial performance index (MPI) or Tei Index, described more than a decade ago, has been well documented in the literature as a prognostic and progression marker for various heart diseases [2–4]; however, in the majority of these studies, MPI was used in patients with combined systolic and diastolic dysfunctions. In isolated left-ventricular (LV) diastolic dysfunction (DD), only a few results have been published [5–7].

One limitation of the conventional Doppler-derived Myocardial Performance Index (PWD-MPI) method is that the measures of time intervals are based on flow-velocity curves and are performed in different cardiac cycles; this method requires several measurements to reduce beat-to-beat variation. An alternative for MPI calculation is the use of the pulsed-wave tissue Doppler imaging-derived myocardial performance index (TDI-MPI), which allows simultaneous measurement of both the diastolic and systolic intervals in the same cardiac cycle, with high diagnostic accuracy in subjects with heart failure and left-ventricular dysfunction [8–10]. As such, the aims of this study were to determine the diagnostic accuracy of PWD-MPI and TDI-MPI and the degree of agreement between these methods in healthy subjects and patients with impaired LV relaxation and a normal ejection fraction and to evaluate the relationship of TDI-MPI to clinical and Doppler echocardiographic parameters.

## Methods

The individuals enrolled in the study were divided into two groups. Group I consisted of 51 consecutive healthy adults volunteers without cardiovascular disease and normal echocardiograms and Group II consisted of 47 consecutive hypertensive patients with normal left-ventricle systolic function and grade-I diastolic dysfunction (DDI patients), defined by the presence of impaired relaxation pattern on Doppler (E/A ratio < 0.8), early diastolic velocity of tissue Doppler imaging (e’) measured at the septal mitral annulus < 8 cm/s and at least two of the following additional criteria: deceleration time of the E wave (DT) > 200 ms, early diastolic velocity of tissue Doppler imaging measured at the lateral mitral annulus < 10 cm/s and average E/e’ (septal and lateral) < 13 [11].

Individuals were eligible if they were 18 years of age or older and had adequate image quality for all echocardiographic measures. The patients had no arrhythmias, left-bundle-branch blocks, pacemakers, myocardial diseases, left ventricular systolic dysfunctions, moderate or severe valvular dysfunctions, or severe health conditions or symptoms during the study.

### Doppler and echocardiographic examination

A comprehensive echocardiogram was performed in all individuals. Cardiac-chamber measurements were performed according to the recommendations of the European Association of Echocardiography and American Society of Echocardiography [12]. Left ventricle mass, relative wall thickness (RWT), fractional shortening and ejection fraction with the modified Simpson’s rule were measured. The mitral inflow-velocity pattern was recorded from the apical four-chamber view with the pulse-wave Doppler sample volume positioned at the tips of the mitral leaflets. The peak velocities of E and A waves, E/A ratio, and deceleration time (DT) were measured. The LV outflow-velocity curve was recorded from the apical long-axis view with the pulsed-wave Doppler sample volume positioned just below the aortic valve.

Tissue Doppler imaging was obtained from the apical four-chamber view, with the sample volume placed at septal and lateral sides of the mitral annulus. Analysis was performed for the peak systolic annular velocity (S), early mitral annulus diastolic velocity (e’), late diastolic velocity (a’) and e’/a’ ratio. With the mitral inflow-velocity curve and the e**’** velocity obtained from the septal and lateral sides of the mitral annulus, the E/e’ ratios and average E/e’ (septal and lateral) were also calculated. Images were stored digitally and measured Qoff-line. The Doppler tracings were obtained at 100 mm/s, and the measures were calculated from an average of five consecutive cardiac cycles.

### MPI calculations

Conventional MPI was measured with pulse Doppler (PWD-MPI) as described by Tei et al. [13]. Interval *“a”,* from cessation to onset of mitral inflow, corresponds to the sum of the isovolumetric contraction time (ICT), ejection time (ET) and isovolumetric relaxation time (IVRT). Interval *“b”* corresponds to ET measured from onset to cessation of LV outflow tract velocity. The sum of ICT and IVRT (MPI numerator) was obtained by subtracting *b* from *a*. MPI was calculated as (a-b)/b. With simultaneous electrocardiographic (ECG) tracing recorded, isolated ICT and IVRT values were determined indirectly. The IVRT was measured by subtracting the interval *“d”* (time between the ECG R wave peak and cessation of LV outflow tract velocity) from the interval *“c”* (time between the ECG R wave peak and the onset of mitral inflow velocity). The ICT was calculated by subtracting IVRT from the interval *“a-b”*.

**Fig. 1:**
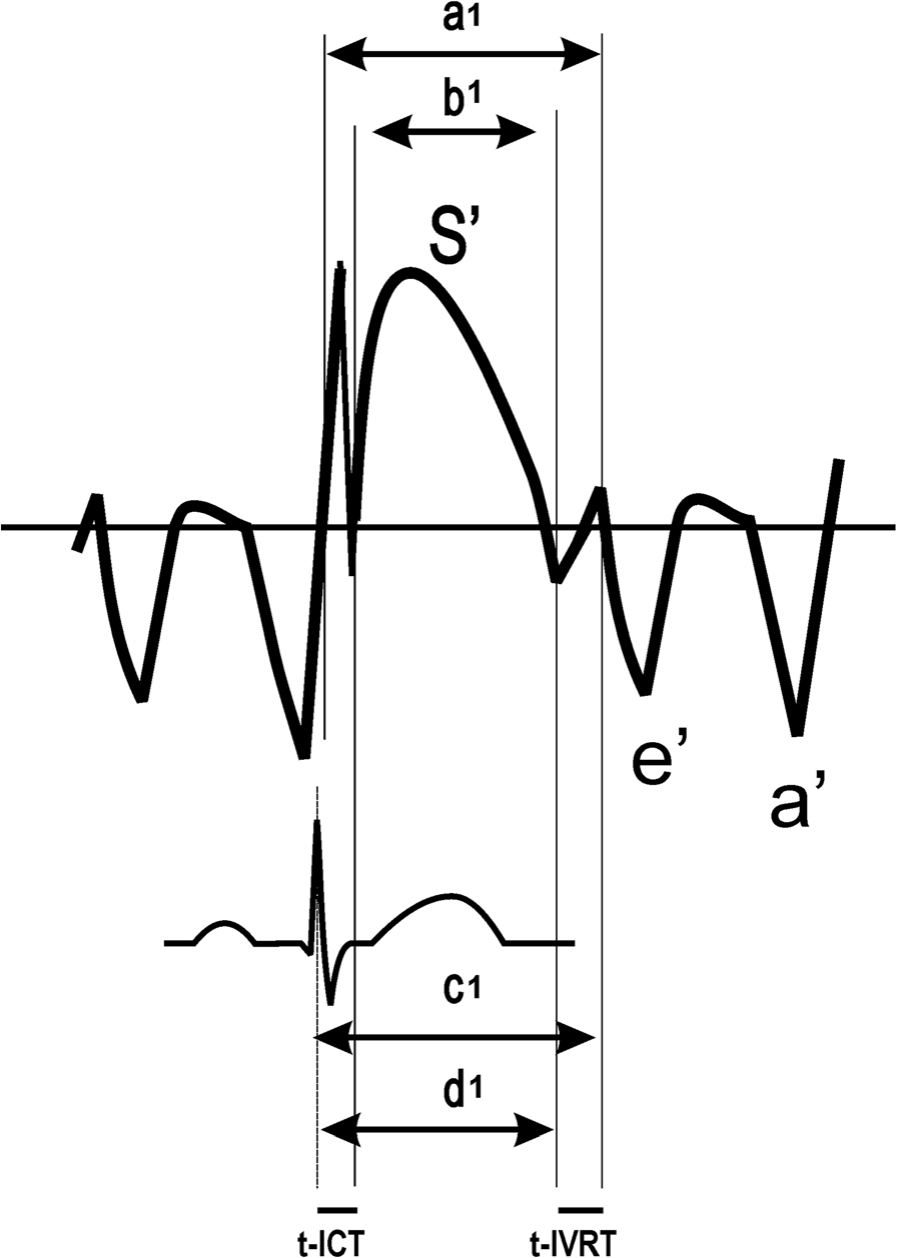
Scheme for measurement of time intervals used to calculate the tissue Doppler-derived myocardial performance index (TDI-MPI): ***a***, time from cessation of the a’ wave to the onset of the e’ wave; ***b***, the duration of the **S** wave; **c**, interval between the **R** wave and onset of the **e**’ wave; **d**, interval between the **R** wave and cessation of the **S** wave; ICT, tissue Doppler-derived isovolumetric contraction time; IVRT

To calculate the myocardial performance index by tissue Doppler imaging (TDI-MPI), time intervals were measured from the septal mitral annulus, as demonstrated in Fig. 1. The interval “a1” (time from cessation of the a’ wave to onset of the e’ wave) corresponds to the sum of tissue Doppler-derived isovolumetric contraction (t-ICT) and relaxation (t-IVRT) times and “b1”, the tissue Doppler-derived ejection time (t-ET), which corresponds to the duration of the S wave (t-ET). The TDI-MPI was calculated as (a1-b1)/b1; t-IVRT was calculated by subtracting the interval “d1”, the time between the ECG R wave, and the cessation of the S wave from the interval “c1”, the time between the R wave, and the onset of e’; t-ICT was calculated by subtracting t-IVRT from (a1- b1).

### Statistical analysis

Data were analyzed with Statistical Package for the Social Sciences (SPSS, Chicago, IL, United States) version 17.0 and were expressed as the mean value ± SD. Kolmogorov-Smirnov normality test was used to evaluate the distribution of the variables. All measurements between two groups were compared using an unpaired Student’s *t*-test, and for variables with a non-normal distribution, the non-parametric Mann-Whitney test was used. The relationship of TDI-MPI with clinical and continuous echocardiographic variables was assessed by the Pearson correlation. Subsequently, significantly correlated variables were further analyzed by multiple linear regression. Bland-Altman plots were used to show the intervals of agreement between the methods; to evaluate the clinical relevance of the differences, a paired Student’s t-test was used to compare TDI-MPI and PWD-MPI in each group. Receiver operating characteristic (ROC) curves were generated, and the areas under the curves (AUC) were calculated with standard errors and 95% confidence intervals to determine optimal diagnostic cutoff values of PWD-MPI and TDI-MPI for the diagnosis of diastolic dysfunction. A 2 × 2 classification table was used to calculate the accuracies, sensitivities, specificities, positive predictive values, negative predictive values, and likelihood ratios of PWD-MPI and TDI-MPI as predictors of diastolic dysfunction.

Intra- and inter-observer reproducibility was assessed in fifteen randomly subjects for TDI-MPI and PWD-MPI. To test the interobserver variability, the measurements were performed off-line from digitally stored images by a second observer who was unaware of the results. The intra-class correlation (ICC) and the mean percentage error, derived as the absolute difference between the two measurements divided by the mean value of the two observations, were calculated to measure the variability. For all tests, a difference was considered significant at *P* < 0.05.

## Results

Demographic data, medication status, general echocardiographic findings and results of the statistical analysis are shown in Table 1. Diastolic function measured by Doppler flow velocities and tissue Doppler and the analyzed time intervals are shown in Table 2. In DDI patients, the TDI-MPI was positively correlated with the diastolic thickness of the interventricular septum (*r* = 0.37, *P* = 0.01), left ventricle posterior wall (*r* = 0.30, *P* = 0.03), left ventricle mass (*r* = 0.39, *P* = 0.03), E/e’ septal (*r* = 0.36, *P* = 0.01) and E/e’ (average; *r* = 0.31, *P* = 0.03) and negatively correlated with S septal (*r* = − 0.38, *P* = 0.009) e’ septal (*r* = − 0.41, *P* = 0.014) and e’/a’ septal (*r* = − 0.36, *P* = 0.014). The relationship of TDI-MPI with age, body surface area, heart rate, systolic and diastolic blood pressures, left atrial diameter, LV diastolic and systolic diameters, LV ejection fraction, and DT did not reach statistical significance. After multiple regression analysis, only the left ventricle mass was still significant independent predictor of TDI-MPI (Table 3).

**Table 1:** Clinical Profile and General Doppler Echocardiographic Findings

	Control *n* = 51	DDI Patients *n* = 47	95% IC	*P*
Age (yr)	38 ± 10	65 ± 10	23 to 31	< 0.001
Male/female	28/23	20/27	0.86 to 1.9	0.23
Body surface area (m^2^)	1.76 ± 0.18	1.68 ± 0.16	0.011 to 0.15	0.051
Hypertension	–	47 (100%)		
Diabetes	–	8 (17%)		
Smoking	–	5 (11%)		
Dyslipidemia	–	26 (55%)		
Heart rate (bpm)	66 ± 7.5	71 ± 8.6	2.62 to 8.9	< 0.001
SBP (mmHg)	113 ± 16	154 ± 22	33 to 48	< 0.001
DBP (mmHg)	67 ± 8.1	83 ± 13	10 to 19	< 0.001
Aortic root (mm)	31 ± 2.9	33 ± 3.6	1.3 to 4.0	< 0.001
Left atrium (mm)	30 ± 2.2	34 ± 4.0	2.7 to 5.3	< 0.001
LVDd (mm)	46 ± 2.9	47 ± 3.0	0.52 to 2.7	0.004
LVSd (mm)	29.4 ± 2.5	30 ± 2.8	−0.43 to 1.7	0.24
Septal thickness (mm)*	9.0 (7–10)	12.0 (12–14)		< 0.001
RWT (cm)	0.40 ± 0.03	0.52 ± 0.05	0.10 to 0.13	< 0.001
LV mass	141 ± 31	226 ± 46	70 to 101	< 0.0001
LV mass index (g/m^2^)	79 ± 12	134 ± 23	48 to 62	< 0.001
Fractional shortening (%)	36 ± 3.5	36.4 ± 4.3	−0.77 to 2.3	0.52
Ejection fraction (%)	65 ± 4.6	66 ± 5.4	−1.02 to 3.11	0.33
Medication				
ACE inhibitors/ARBs		28 (60%)		
Diuretics		20 (43%)		
Beta blockers		15 (32%)		
Calcium-channel blockers		9 (19%)		
Statins		17 (36%)		

DDI, grade-I diastolic dysfunction patients; SBP, systolic blood pressure; DBP, diastolic blood pressure; LVDD, left ventricular diastolic diameter; LVSD, left ventricular systolic diameter; PWT, posterior-wall thickness; RWT, relative wall thickness; ACE, angiotensin-converting enzyme; ARB, angiotensin-receptor blocker

Data expressed as mean ± standard deviation or median and 25-75th interquartile range

* Mann-Whitney test

**Table 2:** Doppler Measurements in Both Study Groups (mean ± SD)

	Control	DDI Patients	95% IC	*p*
E wave (m/s)	0.77 ± 0.16	0.62 ± 0.14	0.091 to 0.21	< 0.001
A wave (m/s)	0.47 ± 0.09	0.82 ± 0.15	−0.40 to - 0.30	< 0.001
E/A ratio	1.67 ± 0.36	0.76 ± 0.10	0.80 to 1.0	< 0.001
DT (ms)	164 ± 17	250 ± 38	−97.6 to −74.1	< 0.001
S septal (cm/s)	7.8 ± 1.2	6.3 ± 1.2	0.99 to 1.9	< 0.001
e’ septal (cm/s)	10 ± 1.6	5.1 ± 1.3	4.6 to 5.7	< 0.001
a’ septal (cm/s)	9.1 ± 1.3	10 ± 1.4	−1.5 to - 0.43	< 0.001
e’/a’ septal	1.1 ± 0.24	0.51 ± 0.12	0.56 to 0.72	< 0.001
E/e’ septal	7.7 ± 1.5	13.1 ± 4.6	−6.8 to −4.1	< 0.001
ET (ms)	308 ± 19	308 ± 27	−0.15 ± 4.6	0.98
IVRT (ms)	80 ± 17	105 ± 21	−32 to − 17	< 0.001
ICT (ms)	35 ± 17	32 ± 19	− 4.7 to 9.5	0.51
PWD-MPI	0.37 ± 0.08	0.45 ± 0.11	− 0.12 to − 0.036	< 0.001
S lateral (cm/s)	11 ± 2.3	9.3 ± 2.1	0.39 to 2.2	0.006
e’ lateral (cm/s)	15 ± 3.2	8.3 ± 2.6	6.0 to 8.5	< 0.001
a’ lateral (cm/s)	9.8 ± 2.2	13.6 ± 3.5	−5.0 to −2.7	< 0.001
e’/a’ lateral	1.7 ± 0.55	0.62 ± 0.19	0.87 to 1.2	< 0.001
E/e’ lateral	5.2 ± 1.4	8.3 ± 3.6	−4.2 to − 2.0	< 0.001
E/e’ average	6.4 ± 1.4	10.8 ± 3.8	−5.5 to − 3.2	< 0.001
t-ET (ms)	317 ± 20	314 ± 28	− 6.6 to 13.2	0.51
t-IVRT (ms)	76 ± 19	109 ± 27	− 43 to − 24	< 0.001
TDI-MPI	0.40 ± 0.09	0.49 ± 0.14	− 0.13 to − 0.035	< 0.001

DDI, grade-I diastolic dysfunction patients; DT, deceleration time of the E wave; S, peak systolic annular velocity; e’, early mitral annulus diastolic velocity; a’, late diastolic velocity; E/e’, relationship between the velocity of the early mitral filling E wave and the e’ wave; ET, ejection time; ICT, isovolumetric contraction time; IVRT, isovolumetric relaxation time; MPI, myocardial performance index; t-ET, tissue Doppler-derived ejection time; t-IVRT, tissue Doppler-derived isovolumetric relaxation time; t-ICT, tissue Doppler-derived isovolumetric contraction time; TDI-MPI, pulsed-wave tissue Doppler-derived myocardial performance index

**Table 3:** Univariate and Multiple Regression Analysis of TDI-MPI

	Univariate Analysis		Multiple Analysis	
	r	*P*	*β*	*P*
LV mass	0.39	0.006	0.325	0.031
S septal	- 0.38	0.009	- 0.332	0.058
e’ septal	- 0.41	0.004	0.092	0.78
e’/a’ septal	- 0.36	0.014		
E/e’ septal	0.36	0.012		

*E* early mitral filling wave, *S* peak systolic annular velocity, *e* early mitral annulus diastolic velocity, *a* late diastolic velocity

*R*^*2*^ = 0.304; Adjusted *R*^*2*^ = 0.219; standard error of estimate = 0.124

β = Standardized coefficient; *r* = Pearson correlation

### Diagnostic value of MPI for evaluation of LV diastolic function

**Fig. 2:**
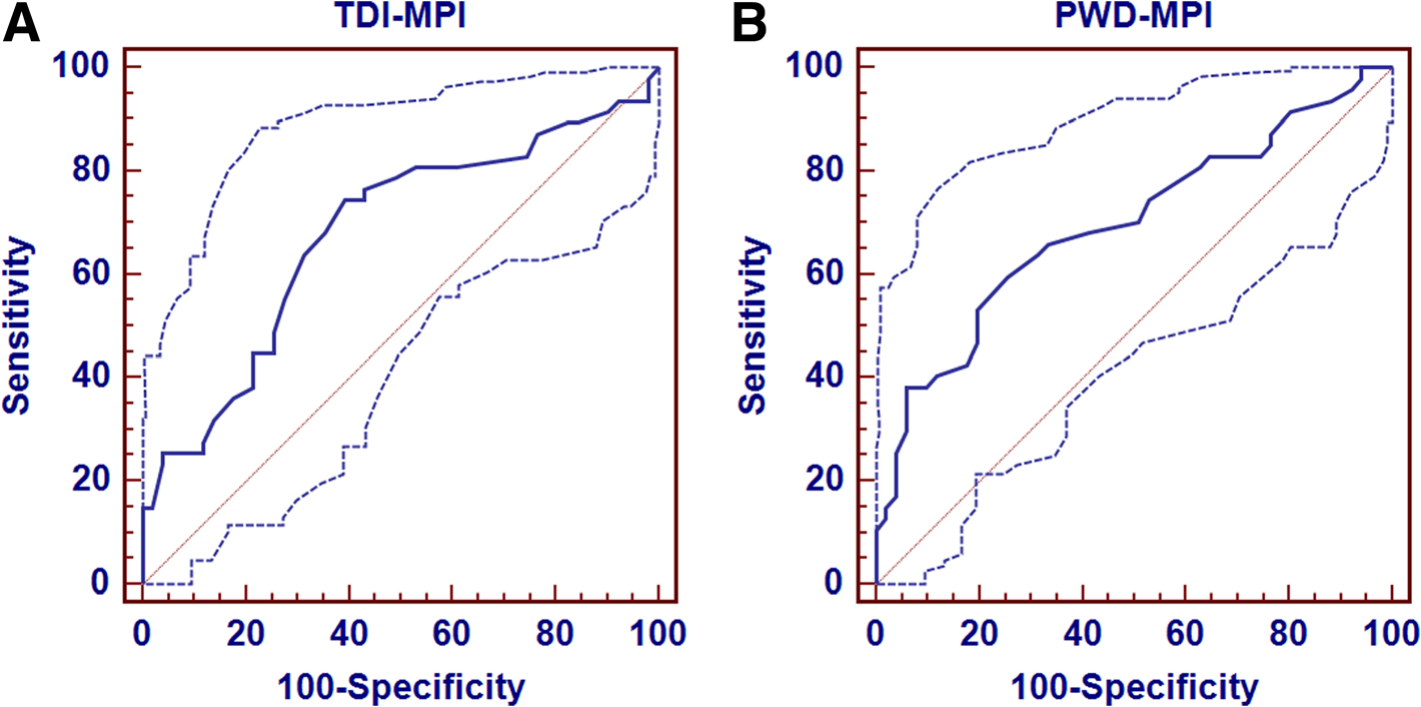
The areas under the ROC curves and the corresponding 95% confidence intervals for (**a**) tissue Doppler-derived myocardial performance index (TDI-MPI) and (**b**) conventional Doppler-derived myocardial performance index (PWD-MPI)

TDI-MPI was significantly higher in the group with diastolic dysfunction compared to those with normal function; the increase was caused by the prolongation of t-IVRT without significant variation in tissue Doppler-derived isovolumetric contraction time and ejection time (Table 2). The AUC of the ROC curve was 0.68 ± 0.06 (95% confidence interval [CI], 0.57 to 0.79, *P* = 0.002; Fig. 2**a**). With a TDI-MPI cut-off value > 0.42, patients with diastolic dysfunction were identified with a sensitivity of 74% (95% CI, 60 to 86%) and a specificity of 61% (95% CI, 46 to 74%). TDI-MPI correctly classified diastolic dysfunction in 35 of 47 subjects and normal diastolic function in 31 of 51 subjects, for false-negative and -positive rates of 26 and 39%, respectively. The prevalence, accuracy, sensitivity, specificity, positive predictive value, negative predictive value, positive likelihood ratio, and negative likelihood ratio are presented in Table 4.

**Table 4:** Measures of TDI-MPI and PWD-MPI for the diagnosis of diastolic dysfunction

	TDI-MPI (Cut-off point > 0.42)	PWD-MPI (Cut-off point > 0.40)	*P*
Accuracy (%; 95% CI)	67 (58 to 76)	66 (57 to 75)	0.91
Sensitivity (%; 95% CI)	74 (60 to 86)	64 (50 to 76)	0.37
Specificity (%; 95% CI)	61 (46 to 74)	69 (55 to 80)	0.53
PPV (%; 95% CI)	64 (50 to 75)	65 (51 to 77)	0.91
NPV (%; 95% CI)	72 (57 to 83)	67 (54 to 78)	0.75
(+) LLR (95% CI)	1.90 (1.30 to 2.8)	2.0 (1.3 to 3.2)	
(−) LLR (95% CI)	0.42 (0.25 to 0.72)	0.53 (0.35 to 0.80)	

*MPI* myocardial performance index, *PPV* positive predictive value, *NPV* negative predictive value; (+) *LLR* positive likelihood ratio; (−)*LLR* negative likelihood ratio

PWD-MPI was significantly higher in the group with diastolic dysfunction (*P* < 0.001) compared to those with normal function. This increase was also caused by the prolongation of IVRT without significant variation in isovolumetric contraction time and ejection time (Table 2). The AUC of the ROC was 0.70 ± 0.05 (95% CI, 0.59 to 0.80, *P* < 0.001) (Fig. 2**b**). With an MPI cut-off value > 0.40, patients with diastolic dysfunction were identified with a sensitivity of 64% (95% CI, 49 to 77%) and a specificity of 69% (95% CI, 54 to 81%). PWD-MPI correctly classified diastolic dysfunction in 30 of 47 subjects and normal function in 35 of 51 subjects, for false-negative and -positive rates of 31 and 36%, respectively. The accuracy, sensitivity, specificity, positive predictive value, negative predictive value, positive likelihood ratio, and negative likelihood ratio are presented in Table 4.

In Group 2, 13 patients (28%) had E/e’ (average) ≥ 13, suggesting increased LV filling pressures [11]. The TDI-MPI was significantly higher in this subgroup compared to the control group as consequence of the isolated prolongation of t-IVRT. The AUC of the ROC was 0.79 ± 0.07 (95% CI, 0.65 to 0.92, *P* = 0.001). With a TDI-MPI cut-off value > 0.42, patients with diastolic dysfunction and high filling pressures were identified with an accuracy of 67% (95% CI, 55 to 77%), sensitivity of 77% (95% CI, 50 to 92%), specificity of 65% (95% CI, 51 to 76%), positive predictive value of 36% (95% CI, 21 to 54%), negative predictive value of 92% (95% CI, 78 to 97%), positive likelihood ratio of 2.18 (95% CI, 1.4 to 3.5) and negative likelihood ratio of 0.36 (95% CI, 0.13 to 0.98).

### Comparison between TDI-MPI and PWD-MPI

**Fig. 3:**
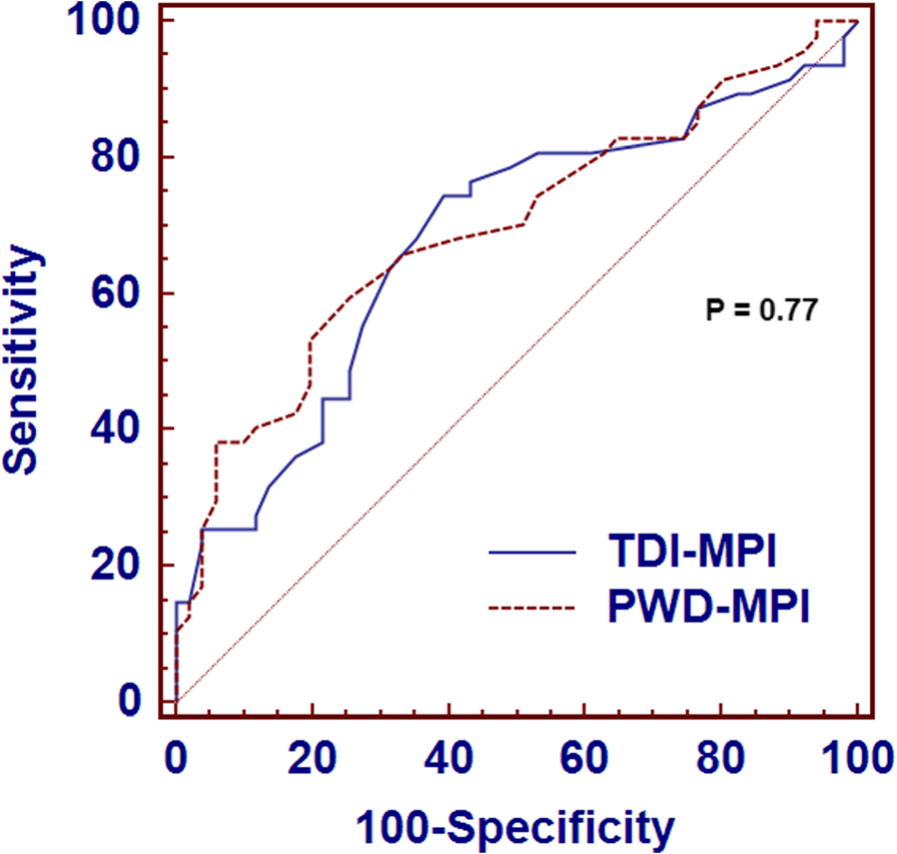
Comparison of the areas under the ROC curves for tissue Doppler-derived myocardial performance index (TDI-MPI) and conventional Doppler-derived Myocardial Performance Index (PWD-MPI)

**Fig. 4:**
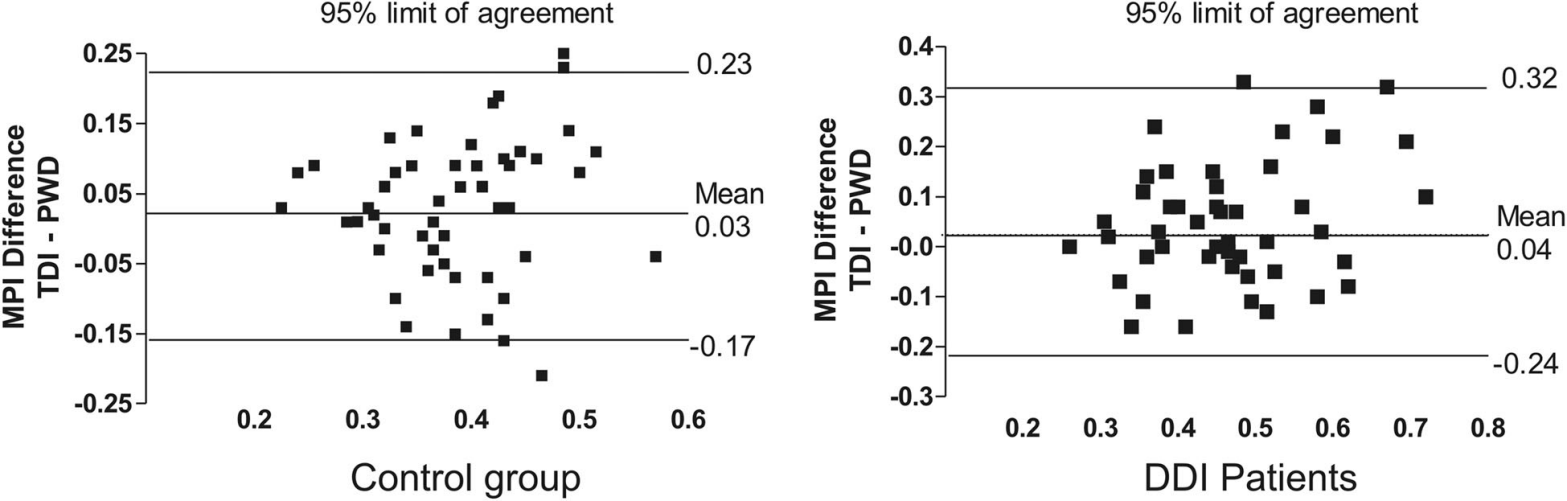
Bland-Altman plot of the differences between TDI-MPI and PWD-MPI for control group and DDI patients

In healthy subjects, the TDI-MPI was higher than the PWD-MPI (0.40 ± 0.09 vs. 0.37 ± 0.08; 95% CI, 0.003 to 0.06; *P* = 0.032); however, the values of these indices were not significantly different in DDI patients (0.49 ± 0.14 vs. 0.45 ± 0.11; 95% CI, − 0.005 to 0.08, *P* = 0.079). In addition, no significant differences were noted between the AUCs of these indices (0.02 ± 0.06, 95% CI, − 0.11 to 0.14, *P* = 0.77; Fig. 3). Although the Bland-Altman plot yielded mean differences between these indices of only 0.03 ± 0.10 and 0.04 ± 0.14 for the control group and DDI patients, respectively, the 95% limits of agreement [LA] were wide (− 0.17 to 0.23 and − 0.24 to 0.32, respectively), suggesting a low level of concordance (Fig. 4).

### Reproducibility

Inter- and intra-observer variability were low: 2.7% ± 0.3 and 5.2% ± 0.1% for PWD-MPI and 1.4% ± 2.3 and 2.2% ± 1.1% for TDI-MPI, respectively. The intra-class correlation coefficients were high: 0.98 and 0.93 (inter-observer) and 0.98 and 0.97 (intra-observer).

## Discussion

This study showed that both PWD-MPI and TDI-MPI were significantly higher in patients with DD and preserved systolic function than in healthy subjects. The increase in both indices was mainly caused by isolated and significant prolongation of the IVRT, the only diastolic component of MPI. The systolic components, isovolumetric contraction time and ejection time, showed no significant difference compared to the control group. IVRT tends to increase in isolated left-ventricular diastolic dysfunction [11] since early diastolic relaxation proceeds more slowly [14]; however, its duration depends on both LV relaxation velocity and the difference between LV end-systolic pressure and left-atrial pressure [11], and occasionally it may shorten or pseudo-normalize with significant increases in left ventricular filling pressures [15]. MPI appears more resistant to pseudonormalization, as increased LV filling pressures are correlated with shorter ejection times [16].

According to previous reports [8, 10, 17], TDI-MPI had higher values than PWD-MPI in both healthy subjects and patients with DD. The limits of agreement between these indices in this study were wide; thus, the two methods cannot be used interchangeably, which is consistent with the results of previous reports [17–19]. Furthermore, these indices had high rates of false-positive and negative results, modest sensitivities and specificities, low positive likelihood ratios and high negative likelihood ratios, showing the low capacity of these methods to differentiate between healthy individuals and those with isolated DD. In clinical practice, it is essential to know the method by which the result of a particular test can be used to predict the risk of a disease; sensitivity and specificity cannot be used for such predictions. Likewise, although the predictive values show the probability of abnormality for the results of a specific test, they depend on the prevalence of the disease in the study sample and can rarely be extrapolated beyond that study. In contrast, likelihood ratios are intrinsic properties of the method, do not depend on the prevalence of the disease, and unlike sensitivity and specificity, which are population characteristics, can be used at the level of the individual patient to calculate the probability of disease; therefore, they represent good alternatives tools for accurate diagnosis [20]. These results coincide with previous research, which revealed poor diagnostic accuracy of conventional MPI in patients with isolated DD [6, 7].

Few studies have related TDI-MPI to diastolic dysfunction. Gaibazzi et al. [8] studied patients with heart failure and found a slight correlation between PWD-MPI and TDI-MPI and high diagnostic accuracy using both the methods for the diagnosis of HF; however, all of those patients had mild to moderate systolic dysfunction, and the authors found no correlation between TDI-MPI and DD. Rojo et al. [18] observed no significant difference between TDI-MPI and PWD-MPI and modest agreement between these methods in patients with recent myocardial infarction; however, the authors did not establish the accuracy of the methods, although most patients had diastolic dysfunction with a normal ejection fractions. Su et al. [21] studied a significant number of heterogeneous patients with DD and reported that TDI-MPI increased with increasing severity of DD and accurately differentiated subjects with pseudonormal filling patterns from those with normal mitral inflow; however, the authors did not determine the diagnostic accuracy in the subgroup of patients with impaired relaxation, although that subgroup contained a considerable number of patients. Baikan et al. [22] proposed that the TDI-MPI index might be superior to the traditional mitral-inflow curves for the assessment of left ventricular diastolic function in patients with acromegaly with preserved systolic function; however, less than half of the 27 patients had DD, and the authors did not assess the sensitivity, specificity, and likelihood ratios of TDI-MPI for the diagnosis of DD. Patel et al. [23] showed a significant increase in TDI-MPI in patients with isolated DD compared to the control group, with good sensitivity, specificity, and likelihood ratios; however, this analysis was conducted on a small subgroup of children with different congenital heart defects. Recently, Kim et al. [24] observed similarly high accuracies in TDI-MPI, E/e′ ratio, and N-terminal pro-brain natriuretic peptide (NT-ProBNP) level for the identification of DD and heart failure with preserved ejection fraction. Moreover, TDI-MPI predicted cardiovascular adverse events with reliability similar to that of the E/e’ ratio and NT-ProBNP level; however, they did not determine the accuracy of TDI-MPI in patients with type-I DD without heart failure.

Among all echocardiographic variables used for the selection and diagnosis of patients with DD in this study, only e′ septal and e´/a′ septal were significantly related to TDI-MPI. After multiple regression analysis, only the LV mass was considered independent predictor of TDI-MPI, which is in accord with a previous study [14]. The correlation between mass and TDI-MPI may be related to the large number of patients with LV hypertrophy, which results in high collagen deposition, decreased LV relaxation and distensibility with a negative impact on ventricular performance [25].

Even patients with type-I DD (E/A < 1) can have increased LV filling pressures [26, 27]. In such cases, although it has been less studied, the E/e′ ratio described by Nagueh et al. [28] and validated by other authors [29, 30] has been a useful tool for the assessment of LV filling pressures. Kasner et al. [30] compared conventional Doppler and TDI with invasive hemodynamic measurements in the estimation of diastolic function and found an average E/A ratio < 1 for patients with DD and increased LV filling pressures with a normal ejection fraction. They identified the lateral E/e’ ratio as the best index for the detection of DD in these patients. Similarly, Kuznetsova et al. [31] described a class of DD characterized by a low E/A ratio and a high E/e′ ratio. They suggested that those patients had a significantly abnormal LV relaxation, such that both left atrial pressure and LV diastolic pressure were elevated. Recently, Johnson et al. [32] classified patients with mild to moderate DD as those who had impaired LV relaxation and showed signs of increased pressure in the left atrium (E/e′ > 15).

In the present study, 13 patients (30%) had an E/e′ ratio (septal and lateral mean) ≥ 13, suggesting increased LV filling pressures, as recommended by the guidelines of the American Society of Echocardiography for the assessment of LV diastolic function [11]. In this subgroup, the area under the ROC curve and the sensitivity and specificity of TDI-MPI were greater than in the overall group, however, a low positive likelihood ratio indicated only a small increase in the probability that high values of TDI-MPI was associated with the presence of DD. Furthermore, unlike Kim et al. [24] we found no correlation between this index and the E/e′ ratio. The accuracy of a diagnostic method depends upon the severity and extent of disease; therefore, considering the low accuracy of the TDI-MPI for patients with mild to moderate DD, as observed in the present study, the utility of this index may be limited for patients with subclinical forms of DD, compromising its use as a marker of global cardiac function.

There are a few limitations in this study that should be considered. Patients were diagnosed with diastolic dysfunction according to the 2009 ASE/EAE echocardiographic recommendations, as these criteria have already been tested in numerous studies [28–32] and have proven to be an important predictor of all-cause mortality in a seminal epidemiologic study [1]. Invasive hemodynamic parameters were not used; thus, it was not possible to determine the influence of hemodynamic factors such as preload, afterload, contractility and systemic vascular resistance in TDI-MPI and its components. However, some studies have shown that TDI-MPI is independent of heart rate, blood pressure and ventricular loading [33, 34]. Left atrial volume index was not evaluated, but considering that this study involved asymptomatic hypertensive patients with a diagnosis of mild diastolic dysfunction, it is unlikely that this index would show relevant additional information. Pulmonary arterial systolic pressure was not included among the measures since the quality of the spectral Doppler tricuspid regurgitation signals was poor or absent and no measurable in 57% of DDI patients and in 61% of subjects of control group. Ischemic heart disease could not be excluded because the patients did not undergo stress testing or coronary angiography. Medical therapy was not homogeneous across patients, which may have influenced these results. The groups were not homogeneous with respect to age, the control group consisted of relatively young and healthy subjects and thus with very low possibility of presenting diastolic dysfunction not detected by the classic methods used in this study preventing the occurrence of type II error. Using age-matched groups could show a lower accuracy of the MPI in the diagnosis of DD, compared to the present study, given the possible positive age-dependency of MPI [8, 35]; although no significant correlation was observed between age and this index in any of the groups in this present study as in previous publications [5, 13, 19]. Also the MPI range for control group and optimal cut-point in the current study was comparable with literature ranges [8, 13, 21, 22], and thus, it is unlikely that the age difference significantly affected these results as well as in other published studies [8, 19]. Moreover, conventional measures of systolic function such as the ejection fraction have some limitations in the assessment of LV contractile properties and may not reflect all aspects of ventricular systole [36].

## Conclusion

MPI measured by conventional pulsed-wave Doppler and tissue Doppler imaging showed poor clinical agreement and low diagnostic accuracy for the diagnosis of diastolic dysfunction in hypertensive patients with normal ejection fraction. The use of these parameters as markers of combined LV systolic and diastolic functions should be reappraised.

## Data Availability

The data and material in the current study are available from the corresponding author on reasonable request.

## Abbreviations

a’: Late diastolic velocity
AUC: Area under the curve
CI: Confidence interval
DD: Diastolic dysfunction
DDI: Grade-I diastolic dysfunction patients
DT: Deceleration time
e’: Early mitral annulus diastolic velocity
ET: Ejection time
ICT: Isovolumetric contraction time
IVRT: Isovolumetric relaxation time
LV: Left-ventricular
MPI: Myocardial performance index
PWD: Pulsed-wave Doppler
ROC: Receiver operating characteristic
RWT: Relative wall thickness
S: Peak systolic annular velocity
TDI: Pulsed-wave tissue Doppler
t-ET: Tissue Doppler-derived ejection time
t-ICT and t-IVRT: Tissue Doppler-derived isovolumetric contraction and relaxation times
